# A case report of partial bowel obstruction as the first symptom of a sizeable adnexal mucinous cystadenoma

**DOI:** 10.1515/iss-2022-0003

**Published:** 2022-06-30

**Authors:** Gerasimia Kirochristou, Stefanos K. Stefanou, Christos K. Stefanou, Stefanos Flindris, Thomas Tsiantis, Periklis Tsoumanis, Kostas Tepelenis

**Affiliations:** Department of Surgery, General Hospital of Filiates, Thesprotia, Greece; Department of Obstetrics and Gynaecology, University Hospital of Ioannina, Ioannina, Greece; Department of Medicine, University of Ioannina, Ioannina, Greece; Department of Surgery, University Hospital of Ioannina, Ioannina, Greece; Department of Endocrine Surgery, Henry Dunant Hospital Center, Athens, Greece

**Keywords:** abdominal discomfort, adnexal masses, mucinous cystadenoma, ovarian cystic formations, partial bowel obstruction, postmenopausal patients

## Abstract

**Objectives:**

Mucinous cystadenomas are among the most common benign adnexal masses. The peak incidence of mucinous cystadenoma appears between the third and fifth decades of life, but rare cases in younger and older women have also been reported. Ovarian cystic formations are usually asymptomatic at early stages, until they grow in size and various compression symptoms appear, such as abdominal discomfort, distention, nausea, vomiting, and increased urination.

**Case presentation:**

This is a case of an 86-year-old woman with partial bowel obstruction due to a sizeable adnexal mass. The patient was submitted to exploratory laparotomy due to intestinal obstruction symptoms, the mass was removed and the final histopathological report indicated a benign mucinous cystadenoma (maximum diameter 25 cm). Physical examination was remarkable due to the large size of the mass. Computed tomography revealed the sizeable abdominal mass in contact with the uterus and the ovaries resulting in bowel compression. Exploratory laparotomy due to bowel obstruction symptoms confirmed the imaging results. The abdominal mass was removed without being ruptured, and total abdominal hysterectomy (TAH) with bilateral salpingo-oophorectomy were done.

**Conclusions:**

Our case report highlights the clinical suspicion that is required for the diagnosis and appropriate treatment of this clinical entity. These tumors are uncommon in postmenopausal women, and when they do appear, they can be difficult to differentiate from cancer.

## Introduction

Adnexal masses arising from the ovaries, fallopian tubes, or surrounding connective tissues are a common gynecologic problem. It is estimated that there is a 5–10% lifetime risk for a suspected ovarian neoplasm for women undergoing surgery [1]. The most common type of benign ovarian tumor in postmenopausal patients is mucinous cystadenoma. The risk of malignant transformation requires a surgical approach or/and regular follow-ups [2].

We record a case of an 86-year-old woman with no remarkable signs and symptoms, who was randomly diagnosed with a sizeable mucinous cystadenoma of the left ovary.

## Case presentation

**Figure 1:**
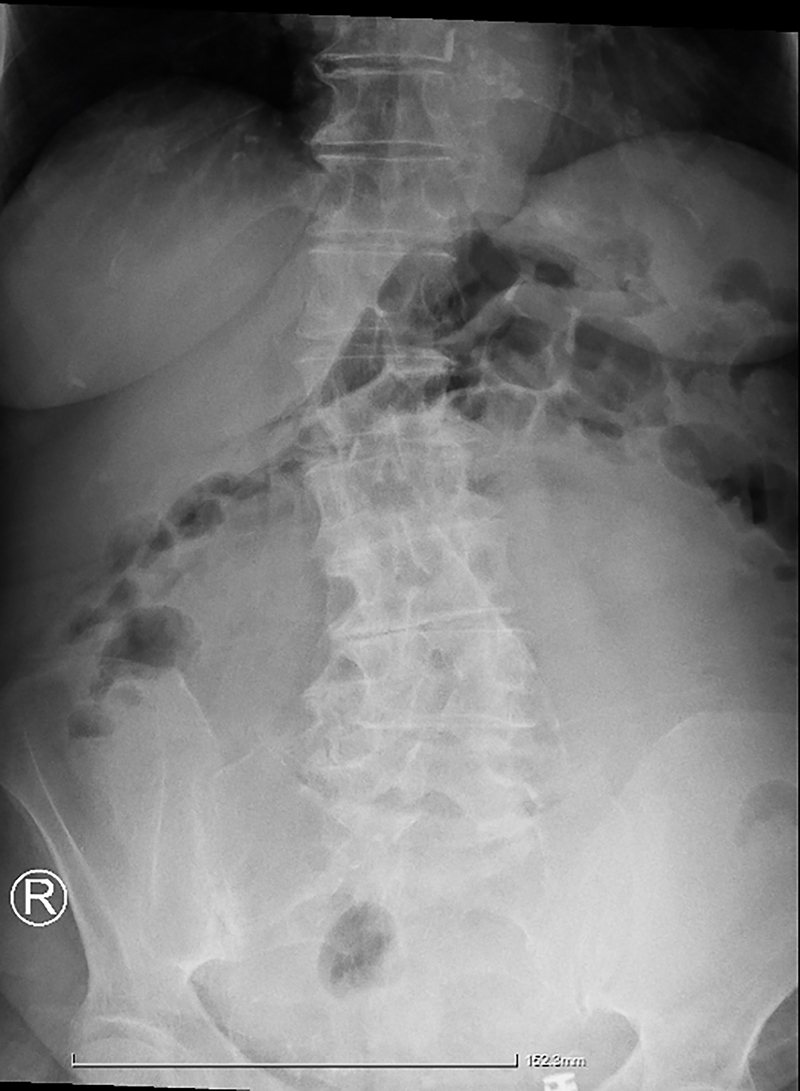
Abdominal X-ray.

An 86-year-old woman presented in the emergency room reporting anorexia, discomfort, flatulence, diffuse abdominal pain and vomiting for three days. Her medical history was free and she had never undergone surgery in the past. During physical examination, abdominal distention and a sizeable palpable mass were identified. Percussion notes were dull, the fluid thrill and shifting dullness was negative and bowel sounds were hard to appreciate due to the large size of the mass. The X-ray strengthened the suspicion of formation, demonstrating a large radiopaque lesion, as the intestine has been repelled laterally (Figure 1). Laboratory testing did not show any significant abnormality (hemoglobin, coagulation, renal, and hepatic profile were within normal limits according to the patient’s age) and CA 125 II was the only slightly elevated tumor marker (85.6 with an average range between 0.0 and 35.0).

**Figure 2:**
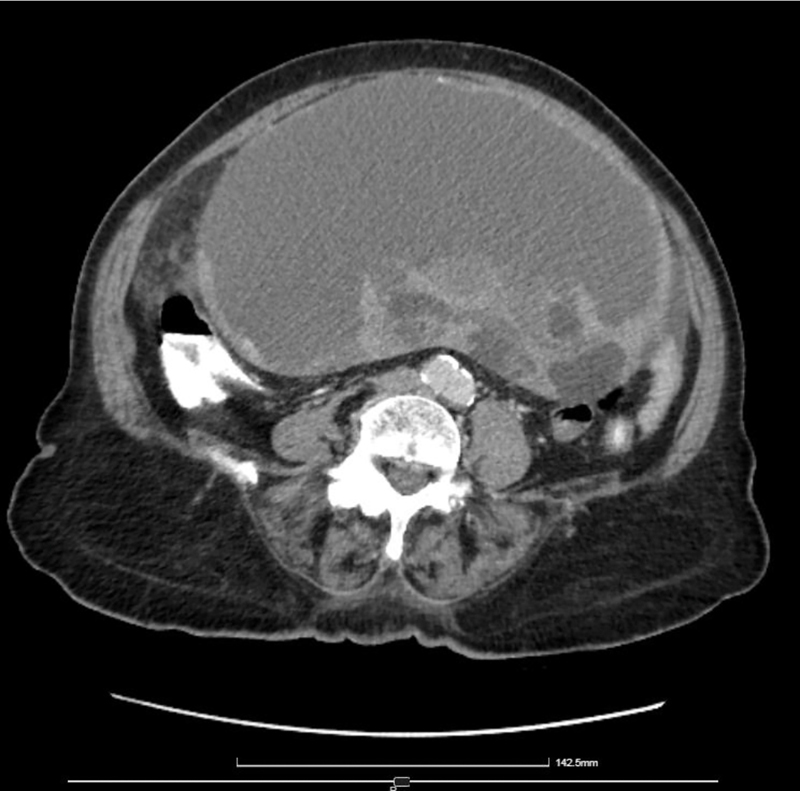
Computed tomography (CT) of the mass.

**Figure 3:**
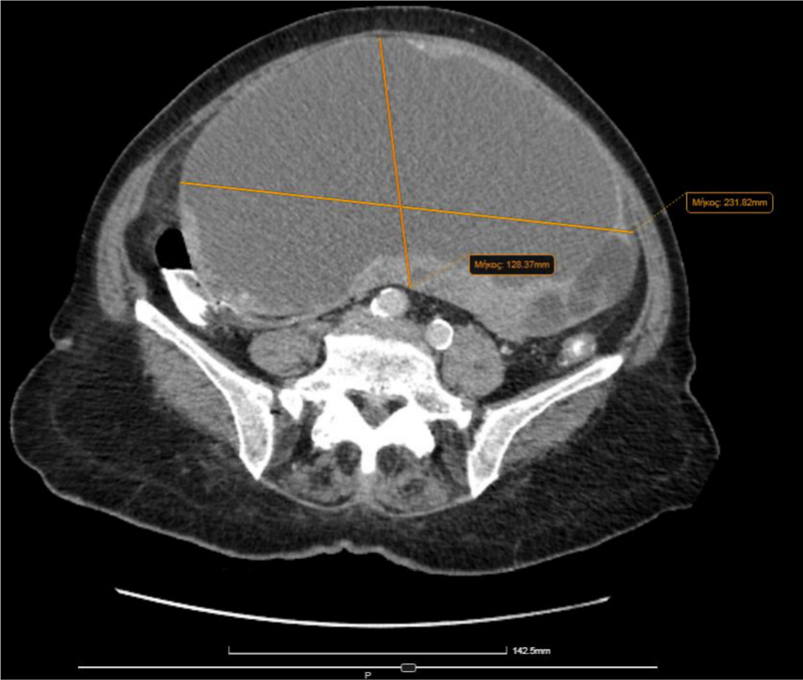
Computed tomography (CT) of the mass.

Computed tomography (CT) scan showed a large abdominal mass (23 × 13 cm) in contact with the uterus and the ovaries, resulting in organ displacement. The findings were attributed to cystic density mass of the left ovary, with fattened diaphragms (Figure 2) and mostly smooth ambit (Figure 3). The uterus was enlarged with lobed fringe and calcified fibroids were present. No enlarged lymph nodes were noticed (no one exceeded 1 cm). Pulmonary parenchyma and mediastinum lymph nodes were normal, too.

**Figure 4:**
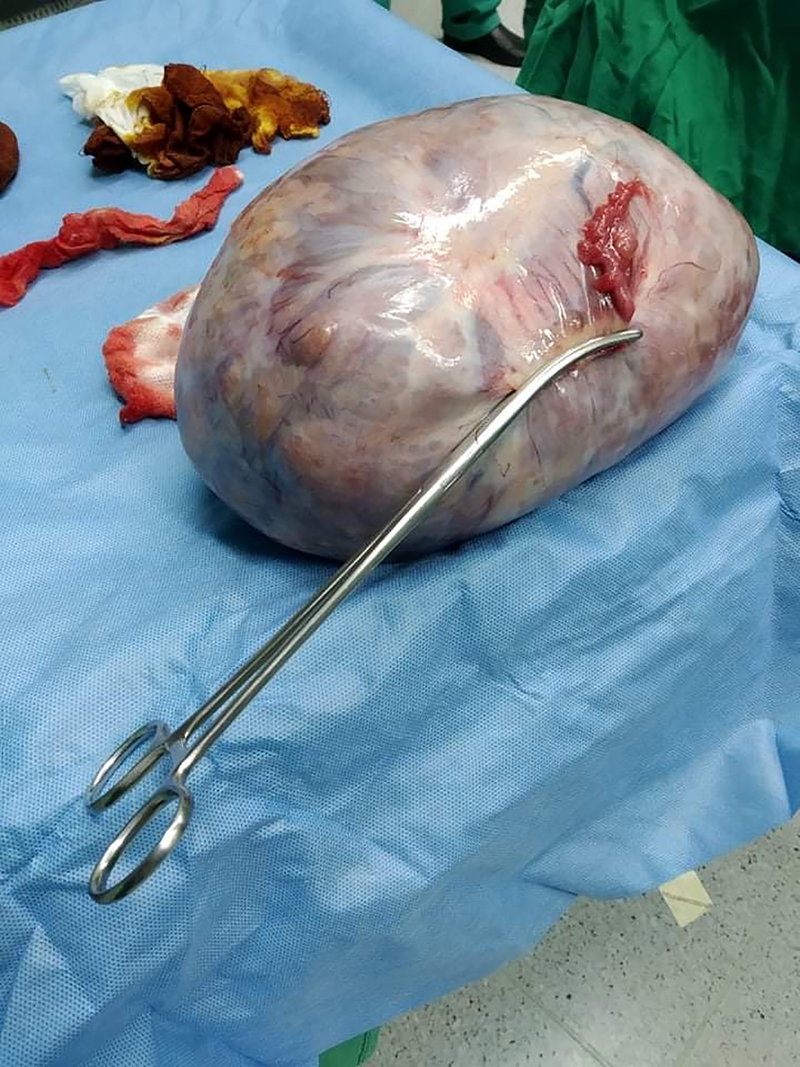
Mass and left ovary after surgical removal.

The patient was submitted to exploratory laparotomy due to bowel obstruction symptoms. The procedure was performed under general anesthesia in a supine position with a midline laparotomy incision. The mass was removed without being ruptured, along with the left ovary (Figure 4). Total abdominal hysterectomy (TAH) and right oophorectomy were done. No lymph node involvement was noted.

The final histopathological report indicated a benign mucinous cystadenoma (maximum diameter 25 cm); therefore, no staging was performed.

The postoperative period was uneventful and the patient was discharged on the 6th postoperative day.

## Discussion

Adnexal masses arising from the ovaries, fallopian tubes, or surrounding connective tissues are a common gynecologic problem. It is estimated that there is a 5–10% lifetime risk for a suspected ovarian neoplasm for women undergoing surgery. There are a wide variety of mass types and they may affect females of all ages, from fetuses to older adults. Pathology in this area may also arise from the uterus, bowel, retroperitoneum, or metastatic disease from another site, such as the gastrointestinal tract or breast [1].

Ovarian masses include physiologic cysts (follicular or corpus luteum), benign neoplasms (such as endometriosis and cystic teratoma/dermoid cyst) and ovarian cancer or metastatic disease from a nonovarian primary tumor. Fallopian tube masses include ectopic pregnancy, hydrosalpinx, and fallopian tube cancer. Apart from these two categories, adnexal masses can derive from nearby structures, too (paratubal or paraovarian cysts, tubo-ovarian abscesses, and broad ligament leiomyomas) [3]. Adnexal masses’ etiology differs by age and reproductive status because some of them are reproductive hormones depended (Table 1) [1].

**Table 1:** Etiology of adnexal masses.

A) Fetuses–children–adolescents	B) Reproductive age	C) Postmenopausal patients	D) Nongynecologic masses
Follicular ovarian cysts	Functional/physiologic cyst	Cystadenoma	Metastatic disease
Physiologic cysts	Corpus luteal cyst	Paraovarian cyst	Abdominopelvic abscess
Cancer (≤5%)	Theca lutein cyst	Hydrosalpinx	Urinary tract masses
	Polycystic ovaries		
	Endometrioma	**Malignant 30%**	
	Uterin leiomyoma	*Epithelial neoplasms*	
	Mature teratomas		
	Serous cystadenoma		
	Mucinous cystadenoma		
	Corpus luteum pregnancy		
	Ectopic pregnancy		
	Tubo-ovarian abscess		
	Hydrosalpinx		
	Cancer (6–11%)		

Adnexal masses may be asymptomatic for years and are incidentally diagnosed by ultrasonography for another cause. Especially ovarian cysts (more common in young females) disappear spontaneously without signs and symptoms. Abdominal discomfort may be present when they are large and increased urination in case of pressure on the bladder. Signs and symptoms may also include: pelvic pain, dysmenorrheal, dyspareunia, nausea, vomiting, fullness, and heaviness in the abdomen, and frequency and difficulty emptying the bladder. Acute, severe pain may be the first sign of a complicated ovarian cyst, hemorrhage, torsion, infarction, or rupture [1]. When symptoms and findings that indicate an adnexal mass are present, pelvic examination and imaging is mandatory.

In postmenopausal patients ovaries are not normally palpated, so the identification of a palpable ovary during the physical examination should raise the suspicion of an ovarian or tubal tumor, and further examination with laboratory testing (to evaluate for malignancy or hormonal activity) and pelvic imaging are required. The physician should never forget the abdominal examination for distention and ascites and/or an abdominal mass. When these findings coexist the diagnosis of malignancy is almost certain [3].

The first diagnostic instrument is pelvic ultrasound, as it is less expensive than other imaging techniques but its diagnostic accuracy is similar. In most patients, both a transvaginal (better resolution) and a transabdominal (better tolerated) approach should be applied. Magnetic resonance imaging (MRI) is secondarily used to determine if a surgical evaluation is needed and computed tomography (CT) is part of noninvasive staging [1]. CA 125 is the most widely used tumor marker that participates in the initial assessment and follows up (sensitivity: 69–87%, specificity: 89–93%) in postmenopausal women, but it can also be elevated in many benign conditions, such as adenomyosis or endometriosis [4].

To summarize according the ESGO/ISUOG/IOTA/ESGE Consensus Statement on preoperative diagnosis of ovarian tumors, ultrasonography is indicated as a first step in stratifying patients with symptoms that suggest an adnexal mass, as well as those who have an adnexal mass accidentally discovered on imaging. An experienced sonographer should perform any ultrasonographic examination in the case of a suspected ovarian mass. The Consensus also took further steps to distinguish the malignancy and the disease’s extent. The tumor marker profile (CA 125 and CEA, complemented with other markers in specific circumstances) in combination with an expert’s ultrasound assessment can often reveal the precise subtype of malignancy. Diagnosis of the primary tumor can be assessed with MRI and before any scheduled surgery for suspected malignancy, a CT scan of the chest, abdomen, and pelvis is required to rule out secondary malignancies, thromboembolic events, and multifocal intraparenchymal distant metastases that would make resection impossible [5].

Mucinous cystadenoma is a benign cystic ovarian tumor. It arises from the surface epithelium of the ovary and is lined by mucin-secreting epithelium [6]. The peak incidence of mucinous cystadenoma appears between the third and fifth decades of life (45–65 years) [7], but rare cases in younger and older women have also been reported. Benign mucinous adnexal tumors account for 10–15% of all ovarian neoplasms [8]. This kind of tumor can be histopathologically classified as benign, borderline, or low malignant, and invasive. Overall, 80% are benign, 10% are borderline and 10% are malignant. On the other hand, though, serous tumors are more likely to be malignant (60% are benign, 15% are borderline and 25% are malignant) [9].

Ovarian cystic formations are usually asymptomatic at early stages, until they grow in size and various compression symptoms appear. They are usually detected incidentally during a routine gynecological examination or on imaging studies for another reason [8]. Because of the widespread use of ultrasound and the advanced imaging modalities that are now available, the reported incidence of giant ovarian cystadenoma in postmenopausal women is low or relatively unknown [10].

When symptomatic, they may cause vague pelvic or abdominal pain, fullness, gastrointestinal symptoms, and progressive abdominal distention [10, 11]. The patients may refer to dyspnea, early satiety, heartburn, and increased urination, too. All these signs indicate a sizeable mass. Severe complications of ovarian neoplasms, such as torsion, hemorrhage, rupture, or even death, are rare, but have been reported [9]. Although mucinous ovarian masses are a benign growth that repels nearby organs laterally, without infiltrating, if they remain untreated, they can grow to gigantic sizes. The largest reported (in 1963) weighted 148.6 kg [12].

Ultimately, surgical resection remains the elective treatment for a large ovarian mass. The laparoscopic approach is increasingly used because of the shorter recovery and decreased perioperative morbidity. This minimally invasive method is successfully used even in cases of sizeable adnexal formations [1, 4, 13]. However, most surgeons prefer laparotomy for very large masses and those suspicious of malignancy. The ovary should be carefully removed intact without spillage to minimize the risk of recurrence [14]. To reduce the risk of other malignancies, a total hysterectomy and bilateral salpingo-oophorectomy are widely preferred in postmenopausal women, regardless of the histology [8].

## Conclusions

Mucinous cystadenoma is a benign cystic ovarian tumor that can reach a huge size. These tumors are rare in the postmenopausal age group, and when they do occur, they pose a diagnostic challenge to differentiate from malignancy. This case report highlights the clinical suspicion that is required, due to the extended asymptomatic period at the early stages.

## Supplementary Material

Supplementary Material
