# Supplementary material for: A case report of partial bowel obstruction as the first symptom of a sizeable adnexal mucinous cystadenoma
Source: Innov Surg Sci. 2022 Jun 30;7(2):71–5. doi: 10.1515/iss-2022-0003 (PMC9574650; doi:10.1515/iss-2022-0003)
Supplement: Supplementary file 1 — Supplementary Material [file j_iss-2022-0003_suppl.pdf]

Reviewer Assessment

C. Stefanou et al.: A case report of partial bowel obstruction as the first symptom of a sizeable adnexal mucinous cystadenoma

Reviewers' Comments to Original Submission

Reviewer 1: Franzke, Timm

Date received: 24-Mar-2022
Reviewer recommendation: **Accept in present form**
Reviewer overall scoring: **Low**

Assessment Form scores: 5 = High/Yes; 3 = Medium/Adequate; 1 = Low

Is the subject area appropriate for the journal		4			
Does the title clearly reflect the paper's content?		4			
Does the abstract clearly reflect the paper's content		4			
Do the keywords clearly reflect the paper's content?		4			
Does the introduction present the problem clearly?		4			
Are the results/ conclusions justified?			3		
How comprehensive and up-to-date is the subject matter presented?			3		
How adequate is the data presentation?				2	
Are units and terminology used correctly?		4			
Is the number of cases adequate?					1
Are the experimental methods/ clinical studies adequate?				2	
Is the length appropriate in relation to the content?			3		
Does the reader get new insights from the article?					1
Please rate the practical significance.			3		
Please rate the accuracy of methods.				2	
Please rate the statistical evaluation and quality control.				2	
Please rate the appropriateness of the figures and tables.			3		
Please rate the appropriateness of the references.		4			
Please evaluate the writing style and use of language.		4			
Please judge the overall scientific quality of the manuscript.				2	
Are the methods used worthy of reproduction in greater deal?	Yes				
Would you be willing to review a revision of this manuscript?	Yes				

Comments to author: No comments

Reviewer 2: Siggelkow, Wulf

Date received: 29-Mar-2022
Reviewer recommendation: **Return to author for minor modifications**
Reviewer overall scoring: **Medium**

Assessment Form scores: 5 = High/Yes; 3 = Medium/Adequate; 1 = Low

Is the subject area appropriate for the journal			3		
Does the title clearly reflect the paper's content?	5				
Does the abstract clearly reflect the paper's content	5				
Do the keywords clearly reflect the paper's content?	5				
Does the introduction present the problem clearly?	5				
Are the results/ conclusions justified?		4			
How comprehensive and up-to-date is the subject matter presented?		4			
How adequate is the data presentation?		4			
Are units and terminology used correctly?					
Is the number of cases adequate?				2	
Are the experimental methods/ clinical studies adequate?			3		
Is the length appropriate in relation to the content?			3		
Does the reader get new insights from the article?				2	
Please rate the practical significance.			3		
Please rate the accuracy of methods.		4			
Please rate the statistical evaluation and quality control.					
Please rate the appropriateness of the figures and tables.		4			
Please rate the appropriateness of the references.				2	
Please evaluate the writing style and use of language.		4			
Please judge the overall scientific quality of the manuscript.			3		
Are the methods used worthy of reproduction in greater deal?	No				
Would you be willing to review a revision of this manuscript?	Yes				

Comments to author: The present paper by Kirochristou et al. highlights the clinical management of a benign adnexal mass in a case report.The presentation of patients history, diagnostic procedures and the surgical approach is appropriate. Concerning the general risk of malignancy in adexel masses in postmenopausal women ESGO-Guidelines (Timmermanns D, et al. ESGO/ISUOG/IOTA/ESGE-Consensus statement on pre- operative diagnosis of ovarian tumors, Int J Gynecol, Cancer, 2021) should be discussed and presented in the reference-section.

Authors' Response to Reviewer Comments

Date received: 29-Apr-2022

Response to reviewer 2

Thank you for reviewing my case report. I read the Timmermans Consensus statement and I added at the discussion the following:

…. To summarise according the ESGO/ISUOG/IOTA/ESGE Consensus Statement on preoperative diagnosis of ovarian tumors, ultrasonography is indicated as a first step in stratifying patients with symptoms that suggest an adnexal mass, as well as those who have an adnexal mass accidentally discovered on imaging. An experienced sonographer should perform any ultrasonographic examination in the case of a suspected ovarian mass. The Consensus also took further steps to distinguish the malignancy and the disease's extent. The tumor marker profile (CA 125 and CEA, complemented with other markers in specific circumstances) in combination with an expert's ultrasound assessment can often reveal the precise sub-type of malignancy. Diagnosis of the primary tumor can be assessed with MRI and before any scheduled surgery for suspected malignancy, a CT scan of the chest, abdomen, and pelvis is required to rule out secondary malignancies, thromboembolic events, and multifocal intraparenchymal distant metastases that would make resection impossible.

Comments by the Editor-in-Chief to Revised Submission

All reviewer comments were addressed adequately and the manuscript should be published in its present stage.
